# Cross-sectional study of human coding- and non-coding RNAs in progressive stages of *Helicobacter pylori* infection

**DOI:** 10.1038/s41597-020-00636-6

**Published:** 2020-09-08

**Authors:** Sergio Lario, María J. Ramírez-Lázaro, Aintzane González-Lahera, José L. Lavín, Maria Vila-Casadesús, María E. Quílez, Anna Brunet-Vega, Juan J. Lozano, Ana M. Aransay, Xavier Calvet

**Affiliations:** 1 Centro de Investigación Biomédica en Red de Enfermedades Hepáticas y Digestivas (CIBEREHD), Instituto de Salud Carlos III, Madrid, Spain; 2 Digestive Diseases Service, Hospital Universitari Parc Taulí, Institut d’Investigació i Innovació Parc Taulí I3PT, Universitat Autònoma de Barcelona, Sabadell, Spain; 3 Genome Analysis Platform, CIC bioGUNE, Bizkaia Technology Park, Derio, Bizkaia Spain; 4 Bioinformatics Platform, CIBEREHD, Barcelona, Spain; 5 Oncology Service, Hospital Universitari Parc Taulí, Institut d’Investigació i Innovació Parc Taulí I3PT, Universitat Autònoma de Barcelona, Sabadell, Spain; 6 Departament de Medicina, UAB, Sabadell, Spain

**Keywords:** Infection, Pathogens, Diagnostic markers, Transcriptomics

## Abstract

*Helicobacter pylori* infects 4.4 billion individuals worldwide and is considered the most important etiologic agent for peptic ulcers and gastric cancer. Individual response to *H. pylori* infection is complex and depends on complex interactions between host and environmental factors. The pathway towards gastric cancer is a sequence of events known as Correa’s model of gastric carcinogenesis, a stepwise inflammatory process from normal mucosa to chronic-active gastritis, atrophy, metaplasia and gastric adenocarcinoma. This study examines gastric clinical specimens representing different steps of the Correa pathway with the aim of identifying the expression profiles of coding- and non-coding RNAs that may have a role in Correa’s model of gastric carcinogenesis. We screened for differentially expressed genes in gastric biopsies by employing RNAseq, microarrays and qRT-PCR. Here we provide a detailed description of the experiments, methods and results generated. The datasets may help other scientists and clinicians to find new clues to the pathogenesis of *H. pylori* and the mechanisms of progression of the infection to more severe gastric diseases. Data is available via ArrayExpress.

## Abstract (summary)

**Table:** 

Measurement(s)	gene expression • ncRNA_gene • miRNA
Technology Type(s)	Microarray • small RNA sequencing assay
Factor Type(s)	H. pylori infection stage • Intestinal metaplasia
Sample Characteristic - Organism	Homo sapiens

Machine-accessible metadata file describing the reported data: 10.6084/m9.figshare.12349334

## Background & Summary

*Helicobacter pylori* is one of the most successful human bacterial pathogens, infecting 4.4 billion individuals worldwide^1^. Infection can induce gastric pathologies ranging from chronic gastritis in all infected individuals to peptic ulcers (in 15–20% of patients) and gastric cancer (0.5–1% of patients)^2^.

Individual response to *H. pylori* infection is complex and depends on a combination of environmental factors, genetic background, host response and strain virulence^3^. The pathway towards gastric cancer is a sequence of events known as Correa’s model of gastric carcinogenesis, a stepwise inflammatory process from chronic-active gastritis (CAG), atrophy (AT), intestinal metaplasia (IM) and gastric adenocarcinoma^4^.

This study examines gastric clinical specimens representing different steps of the Correa pathway with the aim of identifying the expression profiles of coding- and non-coding RNAs (microRNAs and small RNAs) that may have a role in Correa’s model of gastric carcinogenesis and, potentially, to develop novel clinical biomarkers.

**Fig. 1:**
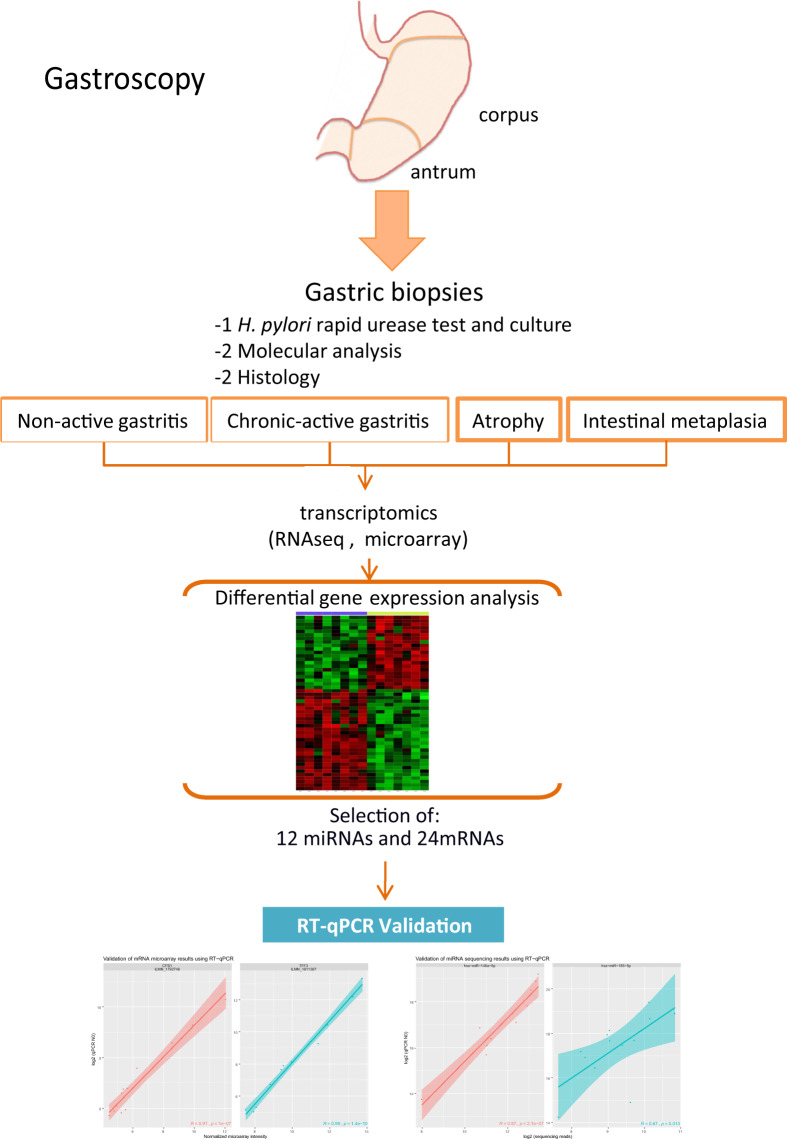
Outline of the experimental design and workflow for this study from biopsy collection to data analysis. During the endoscopy procedure, antrum and corpus biopsies were collected for molecular analysis, rapid urease test and histology. Each specimen was analyzed for *H. pylori*, chronic-active gastritis, atrophy and intestinal metaplasia. Patients with *H. pylori* and neutrophil infiltrate (activity) were classified as chronic-active gastritis (CAG). Patients without activity and negative for *H. pylori* were classified as non-active gastritis (NAG).

RNAseq (for microRNAs and non-coding RNAs) and microarrays (for coding RNAs) were used to screen for differentially expressed genes in gastric biopsies (antrum/corpus). The expression of a selection of genes was confirmed in a validation cohort of patients using quantitative real-time PCR (RT-qPCR). The general study design is illustrated in Fig. 1. Here we provide a detailed description of the experiments conducted, methods used and results generated. The datasets may help other scientists and clinicians to find new clues to the pathogenesis of *H. pylori* and the mechanisms of progression to severe disease states. The transcriptomics data is available in the ArrayExpress database^5^.

## Methods

### Patient selection

The Digestive Service has assembled a collection of samples from dyspeptic patients. The study was undertaken in accordance with the Declaration of Helsinki, with the approval of the ethics committee at our institution (code: 2005511; approval date: 2006/1/11).

**Fig. 2:**
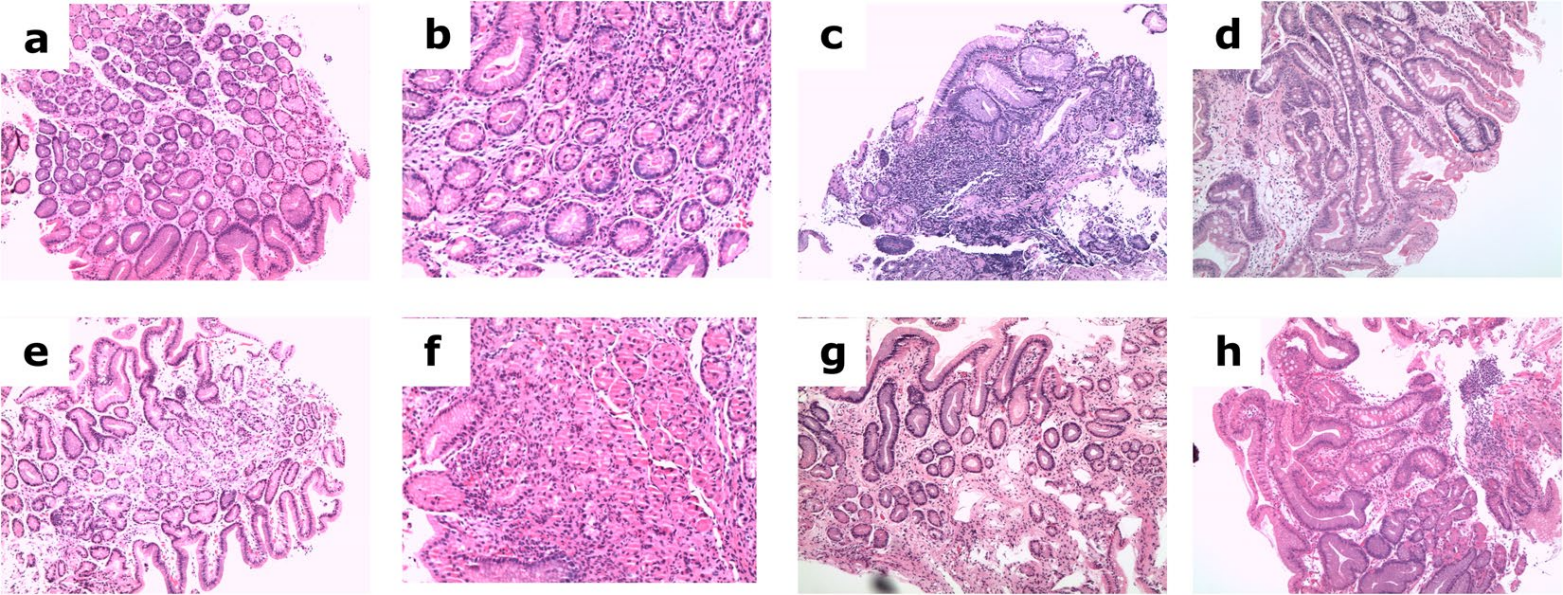
Microscopic images from representative 10x hematoxylin-eosin–stained cases of non-active gastritis (**a, e**), chronic-active gastritis (**b**,**f**), atrophy (**c**,**g**) and intestinal metaplasia (**d**,**h**) are shown for antrum (**a**–**d**) and corpus (**e**–**h**).

At the time the transcriptomic experiments were performed, the collection included samples from 439 patients, enrolled from 2007–2012. The enrolment process was as follows: Dyspeptic patients referred for upper gastrointestinal endoscopy because of dyspepsia were contacted by phone and invited to participate. Those who agreed were instructed not to take antisecretory drugs for two weeks before undergoing the procedure. Exclusion criteria were: patients who were not able to stop antisecretory drugs, those who had received antibiotics in the four weeks before the endoscopy and those with a history of prior treatment for *H. pylori*. Before the endoscopy, a ^13^[C]-urea breath test (UBT) (Cat.No. 654057, UBiTest 100 mg, Otsuka Pharmaceutical Europe Ltd, UK) was administered. During the endoscopy procedure, biopsies were taken for histology, rapid urease testing (RUT, Cat.No. 1100090, JATROX HP test CHR Heim Arzneimittel GmbH, Germany) and molecular analysis (RNAlater, Cat.No AM7021, ThermoFisher, MA, USA). After a positive RUT test, biopsies were plated on Pylori Agar (Cat.No. 413193, bioMérieux SA, Spain) in microaerophilic jars (Jar Gassing System, Don Whitley Scientific Limited, UK). After a maximum of a week, grown *H. pylori* isolates were subcultured on Columbia plates (Cat.No. 43041, bioMérieux) and identified by colony morphology, Gram-negativity and positivity for urease, catalase, and oxidase tests. *VacAs*, *VacAm* and *cagA* virulence factor genes of *H. pylori* were determined by PCR on isolated strains or biopsy samples by using custom locked nucleic acids primers (LNA, Exiqon, Denmark) and SensiMix SYBR Low-ROX Kit (Cat. No. QT625-05, Bioline, UK). Details on *VacAs*, *VacAm* and *cagA* amplification are described in detail elsewhere^6^. For histopathological evaluation, sections were stained with haematoxylin-eosin (Fig. 2) and evaluated for *H. pylori*, CAG, atrophy, intestinal metaplasia, and presence of lymphoid follicles by a pathologist specializing in digestive diseases.

Patients were considered to be *H. pylori* positive when two or more diagnostic tests (RUT, UBT, histology, culture) were positive and /or two or more *H. pylori* virulence PCR assays were positive. Patients with less than two positive diagnostic tests and with less than two positive PCR assays were considered uninfected.

Seventy antral and 26 corpus biopsies from 76 patients were selected. Due to chip restrictions, 2 antral biopsies (B294, B311) were not included in microarray analysis. In 18 cases, antrum and corpus biopsies were paired. The biopsy samples were classified into different groups based on histology: non-active gastritis (NAG, n = 16), chronic-active gastritis (CAG, n = 28), atrophic gastritis (AT, n = 15) and intestinal metaplasia (IM, n = 37). Demographic and clinical characteristics of patients can be found in Online only Table 1.

### RNA extraction and quality control

**Fig. 3:**
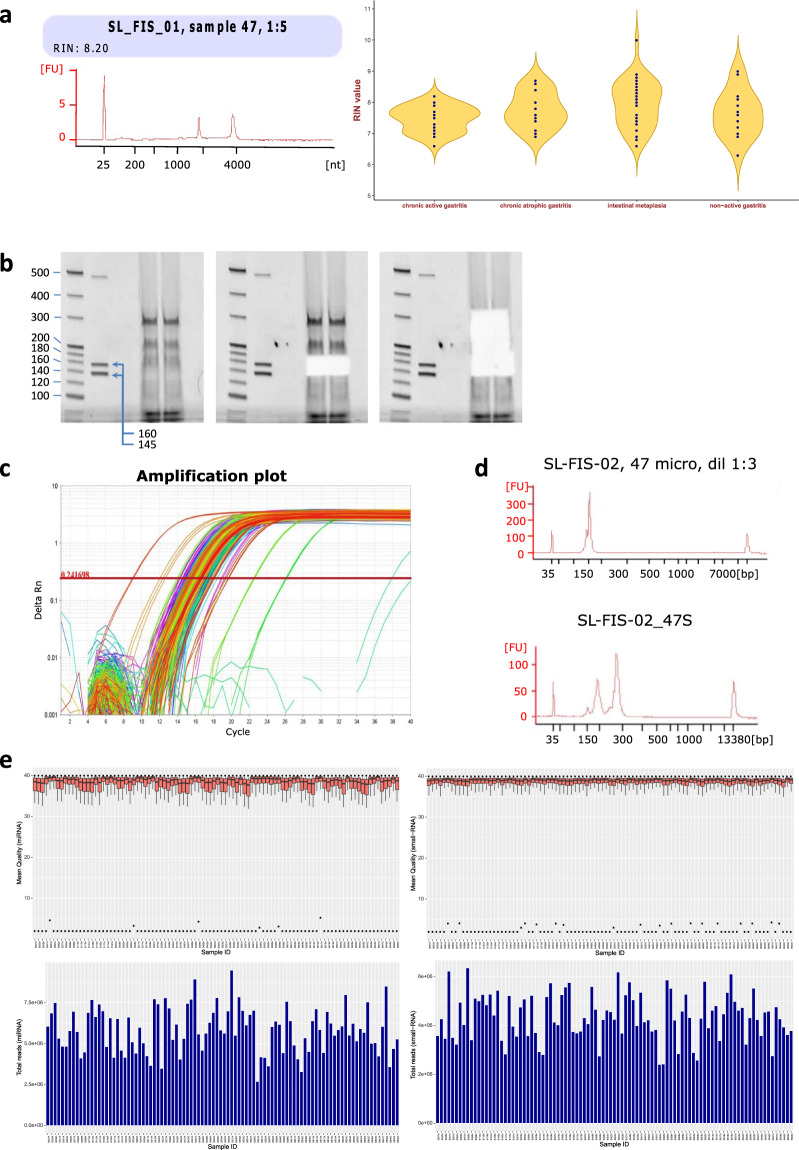
Quality control of the RNA samples, sequencing libraries and sequencing reads. (**a**) Agilent Bioanalyzer electropherogram showing total RNA from sample #47 (*left*) and violin plot of RIN values according to disease state (*right*). (**b**) Library Size Selection by resolution of total RNA on 6% Novex TBE PAGE Acrylamide gels. Original acrylamide gel for sample #47 (*left*), after 145–160 bp miRNA bands (*middle*) and small-RNA fragments (200–300 bp) (*right*) were excised. (**c,d**) Final library QC. Precise library quantification was performed using real-time PCR and size distribution was assessed with Agilent BioAnalyzer High Sensitivity DNA Chips. Upper and lower panels show sample #47 miRNA and small-RNA libraries, respectively. (**e**) Sequencing of the 192 libraries generated over 9.8 × 10^8^ raw reads. Mean library counts were 5.9 × 10^6^ and 4.4 × 10^6^ for miRNAs and small RNAs, respectively. The panels show the distribution of quality scores per base (*upper panels*) and the read count per library (*lower panels*) for both miRNA (*left panels*) and small-RNAs (*right panels*).

Two antrum and two corpus biopsies were used to isolate total RNA. Total RNA was extracted using the mirVana miRNA isolation kit (ThermoFisher, MA, USA) as per the manufacturer’s protocol and stored at −80 °C for downstream analysis. DNase treatment was performed as described in the DNA-free Kit protocol (Cat. No. AM1906, ThermoFisher, MA, USA). Total RNA was quantified with the Qubit® RNA Assay Kit (ThermoFisher, MA, USA). Quality was assessed using Agilent RNA 6000 Nano chips (Cat.No. 5067-1511) on an Agilent 2100 Bioanalyzer (Agilent Technologies, Santa Clara, CA), including calculation of the RNA integrity number (RIN). The RIN score was 7.72 ± 0.6 (Fig. 3).

### mRNA microarrays

Biotin-labeled cRNA samples for hybridization were prepared from 200 ng total RNA using Epicentre TargetAmp Nano-g Biotin-aRNA Labeling Kit for Illumina system (Cat. No. TAN091096; Epicentre, WI, USA). Labeled cRNA was hybridized to the HumanHT-12_V4.0 expression arrays (Cat. No. BD-103-0204; Illumina Inc., San Diego, CA) as described in the protocol/instructions. HumanHT-12 v. 4 Expression arrays were scanned with the iScan system (Illumina Inc., San Diego, CA, USA) and raw data were decoded using GenomeStudio Gene Expression Module (Illumina Inc., San Diego, CA, USA). Intensities were quantile-normalised and differentially detected transcripts were calculated using the Bioconductor *limma* package^7^.

### miRNA and small RNA sequencing

#### TruSeq miRNA and small RNA library preparation

Briefly, 3′ adapter ligation was performed by incubating 1 µg of total RNA of each sample with the adapter for 2 minutes at 70 °C. Then 5′-adapter was added alongside using a truncated T4-RNA ligase 2 (Cat. No. M0351S, New England Biolabs, MA, USA) in an incubation at 28 °C for 1 hour. Half of the ligation product was used for the reverse transcription performed with SuperScript II reverse transcriptase (Cat. No. 18064-014, ThermoFisher, MA, USA) in a thermocycler for 1 hour at 50 °C. Next, enrichment of the cDNA was performed using PCR cycling: 98 °C for 30 secs; 11 cycles of 98 °C for 10 secs, 60 °C for 30 secs and 72 °C for 15 secs; a final elongation of 72 °C for 10 mins, and pause at 4 °C. PCR products were resolved on 6% Novex TBE PAGE gels (Cat. No. EC6265BOX, ThermoFisher, MA, USA). microRNA and Small_Non-coding-RNA fragments between 145–160 and 200–300 bp respectively, were cut from the gel. microRNA and Small_Non-coding-RNA libraries were extracted from polyacrylamide gel with the MinElute gel extraction kit (Cat. No. 28604, Qiagen, Germany) using an adapted protocol, in which gel slices were dissolved in a diffusion buffer (0.5 M ammonium acetate; 10 mM magnesium acetate; 1 mM EDTA, pH 8.0; 0.1% SDS) overnight at room temperature plus 3 hours and 30 min at 50 °C. The libraries were visualized on an Agilent 2100 Bioanalyzer with the Agilent High Sensitivity DNA kit (Cat. No. G2938-90320, Agilent Technologies, Santa Clara, CA) and quantified using quantitative PCR with the Kappa Library Quantification Kit (Master Mix and DNA Standards, Cat.No. KK4824, Roche-Kappa, Basel, Switzerland).

#### Next-generation sequencing (NGS)

The libraries were pooled, and 12pM 12xmicroRNA-libraries and 14pM 12xSmall_Non-codingRNA-library pools were sequenced. Multiplexed libraries were hybridized to flow cells on a cBot Cluster Generation System (Illumina, San Diego, CA, USA) using TruSeq SR Cluster Kit v3-cBot-HS (Cat. No. GD-401-3001; Illumina, San Diego, CA, USA). The clustered flow cells were loaded onto a HiScanSQ sequencer. The sequencing was performed using the TruSeq SBS Kit v3-HS (Cat. No. FC-401-3002; Illumina, San Diego, CA, USA) for 50 cycles.

#### NGS Data analysis

Base calling was performed with the Illumina Real Time Analysis software (RTA, version 1.13.48) and the FASTQ files were generated with CASAVA (version: 1.8.1). Secondary data analysis was done using the sRNAbench package^8^. Briefly, reads were aligned to the human genome (UCSC hg19) using Bowtie 1.1.2^9^. miRNA annotations were obtained from miRBase^10^ (version 21). Sequencing analysis was done by using the sRNAbench package^11^. Briefly, after adapter trimming and unique read grouping, reads were aligned to the human genome (UCSC hg19) using Bowtie^9^ allowing for one mismatch. To provide annotations for RNA elements that mapped to the human genome, miRBase (version 21) for mature and pre-miRNA sequences was used and a matrix of counts were created. To process count and to identify differentially expressed miRNAs we use *edgeR* package^12^.Transcripts were considered differentially expressed provided their edgeR FDR-adjusted P value was < 0.05.

#### Quantitative PCR validation

Twenty-five RNAs were reanalyzed to validate 24 messenger RNAs and 12 miRNAS. The RNAs used were a subset (n = 25) of the aliquots of the same RNA samples we used for sequencing and microarray analysis. Studied genes are summarized in Table 1.

**Table 1:** qPCR Primer assays used for mRNA and miRNA validation.

GENE Symbol	Refseq accession	Detects all variants^(a)^	Exon location^(a)^	Mean PCR efficiency^(b)^	Company and catalogue number
**AGPAT2**	NM_006412(1)	No	4–5	1,86	IDT Hs.PT.58.1470724
**ACTB**	NM_001101(1)	Yes	6–6	1,86	IDT hs.PT.56a.40703009.g
**ANXA13**	NM_004306(2)	Yes	10–11	1,90	IDT Hs.PT.56a.20938889.g
**APOB**	NM_000384(1)	Yes	8–9	1,87	IDT Hs.PT.56a.19389676
**C3**	NM_000064	Yes	27–28	1,90	IDT Hs.PT.56a.2840009
**CDX1**	NM_001804(1)	Yes	1–2	1,89	IDT Hs.PT.58.468499
**CFH**	NM_001014975(1)	No	9–10	1,89	IDT Hs.PT.58.41054235
**CPS1**	NM_001122633(3)	Yes	27–28	1,88	IDT Hs.PT.58.2708374
**CREB1**	NM_134442	No	3–5	1,89	IDT Hs.PT.58.4988504
**CXCR5**	NM_001716(1)	No	1–2	1,89	IDT Hs.PT.56a.1692541
**EIF4G2**	NM_001042559(3)	Yes	3–5	1,91	IDT Hs.PT.58.6917393
**FUT9**	NM_006581	Yes	2–3	1,89	IDT Hs.PT.58.22395619
**HIPK3**	NM_005734(2)	Yes	3–4	1,89	IDT Hs.PT.58.2927056
**HNF4G**	NM_004133(1)	Yes	2–3	1,86	IDT Hs.PT.58.26995600
**IL8**	NM_000584(1)	Yes	3–4	1,82	IDT Hs.PT.58.38869678.g
**KRT20**	NM_019010(1)	Yes	5–6	1,89	IDT Hs.PT.58.39027228
**MEG3**	NR_002766(8)	No	5–10	1,86	IDT Hs.PT.58.25426100
**MMP9**	NM_004994(1)	Yes	3–4	1,84	IDT Hs.PT.58.22814824.g
**MTTP**	NM_000253(1)	Yes	18–19	1,87	IDT Hs.PT.58.94887
**MUC2**	NM_002457(1)	Yes	28–30	1,89	IDT Hs.PT.58.4321237
**POFUT1**	NM_015352(1)	No	6–7	1,78	IDT Hs.PT.58.19361092
**RUNX2**	NM_001024630(3)	Yes	6–7	1,88	IDT Hs.PT.56a.19568141
**SDHA**	NM_004168(1)	Yes	3–4	1,88	IDT Hs.PT.58.41017719
**TFF3**	NM_003226(1)	Yes	1–2	1,89	IDT Hs.PT.58.1814807
**WDR1**	NM_017491(1)	No	4–5	1,88	IDT Hs.PT.58.40308614
**MIR103A1**	NR_029520.1	NA	NA	1,89	Exiqon 204063
**MIR146A**	NR_029701	NA	NA	1,86	Exiqon 204688
**MIR153–1**	NR_029563	NA	NA	1,81	Exiqon 204338
**MIR155**	NR_030784.1	NA	NA	1,90	Exiqon 204308
**MIR182**	NR_029614.1	NA	NA	1,89	Exiqon 206070
**MIR191**	NR_029690.1	NA	NA	1,86	Exiqon 204306
**MIR192**	NR_029578.1	NA	NA	1,91	Exiqon 204099
**MIR196B**	NR_029911.1	NA	NA	1,88	Exiqon 204555
**MIR19B1**	NR_029490.1	NA	NA	1,85	Exiqon 204450
**MIR204**	NR_029621.1	NA	NA	1,89	Exiqon 206072
**MIR215**	NR_029628.1	NA	NA	1,87	Exiqon 204598
**MIR340**	NR_029885.1	NA	NA	1,89	Exiqon 206068

^a^Primer assays targeting all splicing variants were chosen for validation purposes, and when possible, in the same exon where the Illumina probe was positioned.

^b^PCR efficiency was calculated by LinRegPCR software. Using the raw qPCR data, the algorithm computes iteratively a Window-of-Linearity for a specific amplicon and calculates the C_q_ and PCR efficiency for each individual reaction and amplicon.

NA: not applicable.

#### cDNA synthesis

miRNA validation was performed using the miRCURY LNA Universal RT microRNA PCR system (Exiqon, Denmark). miRNAs were reverse transcribed according to the manufacturer’s protocol using 10 ng of total RNA (Cat. No. 203301; miRCURY LNA™ Universal RT microRNA PCR, Polyadenylation and cDNA synthesis kit II). For coding RNAs, 1.0 µg of total RNA was converted into cDNA using PrimeScript RT Reagent Kit (Cat. No. RR037A, Takara, Japan).

#### Quantitative PCR

Coding RNAs were amplified using predesigned PrimeTime 5’ Nuclease Assays (IDT, Iowa, USA) (assay catalog numbers are in Table 1) and PremixExTaq Probe qPCR mastermix (Cat. No. RR390W; Takara, Japan). miRNAs were quantified using predesigned microRNA LNA PCR Primer sets (Exiqon, Denmark) and SensiMix SYBR Low-ROX Kit (Cat. No. QT625-05, Bioline, UK). Amplification was performed in duplicate on a QuantStudio 7 Flex Real-Time PCR System (Applied Biosystems, Foster City, CA, USA) using 384-well plates.

#### qPCR data analysis

The raw PCR data was exported from QuantStudio Real-Time PCR Software v1.2 (Applied Biosystems) onto a RDML^13^ file and imported into LinRegPCR (v2016.1)^14^. LinRegPCR was used to determine PCR efficiencies (E) and to calculate the starting concentration per sample (N_0_). First, the program determines the baseline fluorescence and performs baseline subtraction. Then a Window-of-Linearity for all PCR samples per amplicon is set and then the algorithm determines: the mean PCR efficiency per amplicon (E_mean_), the quantification cycle (C_q_) value per sample and the fluorescence threshold set to determine the C_q_ (N_q_). With these data, N_0_ is calculated using N_0_ = N_q_ / (E_mean_)^Cq^.

## Data Records

Individual miRNA and small-RNA FASTQ files and a tab*-*delimited file for the processed microarray data have been deposited in the ArrayExpress public repository^5^. The accession numbers are: E-MTAB-8890^15^ for miRNAs, E-MTAB-8896^16^ for small RNAs and E-MTAB-8889^17^ for mRNAs. The sample metadata records are provided in Online only Table 1.

## Technical Validation

### Quality control

#### Sample collection

In order to ensure the collection of biopsy tissue samples would provide high-quality results for microbiology, molecular analysis and histology, a two round biopsy protocol was followed. During the endoscopy, a first set of biopsy samples was collected for microbiological (in sterile saline) and molecular analysis (in RNAlater) and a second set were fixed in formalin for histopathological examination. By doing this, we ensured that formalin contamination of biopsy forceps did not interfere with the RUT and *H. pylori* culture. Histological examination was performed by a pathologist specialized in digestive diseases. In order to increase the total RNA yield and because intestinal metaplasia is typically present as small mucosal patches, we isolated RNA from two gastric biopsies per anatomical location. The reason is that the biopsy cores examined by the pathologist are different from the biopsy specimens used for molecular analysis. By using two biopsies, we were more confident that if the pathologist reported intestinal metaplasia in the histology specimens, intestinal metaplasia would also be present in the molecular biology cores. Additionally, two biopsies are the minimum recommended by the Updated Sydney System^18^.

#### RNA processing

Figure 2 shows the quality control procedures used in this study for RNA integrity, library preparation and sequencing.

#### Gene expression validation by qPCR

We used LinRegPCR^14^ for calculating individual and mean PCR efficiencies. Amplicons showed high PCR efficiencies, ranging from 1.78 to 1.91. PCR inhibition can be detected using individual PCR efficiency values. Samples showing PCR efficiencies greater than 5% of the PCR mean efficiency per amplicon were excluded. The algorithm also calculates N_0_. N_0_ is the starting quantity of mRNA or miRNA (expressed in arbitrary fluorescence units). Quantitative N_0_ values have been used in previous publications^19–24^. Determining N_0_ has several advantages over relative quantification. First, the selection of a housekeeping gene is often controversial since the expression of all genes is regulated. Second, the expression of a housekeeping gene varies to a greater or lesser extent under experimental conditions^25^. Third, to solve this issue a quantitative PCR approach with a correction factor according to the starting amount of RNA used in the reverse transcription has been recommended (i.e. µg of RNA)^26^ instead of relative quantification.

**Fig. 4:**
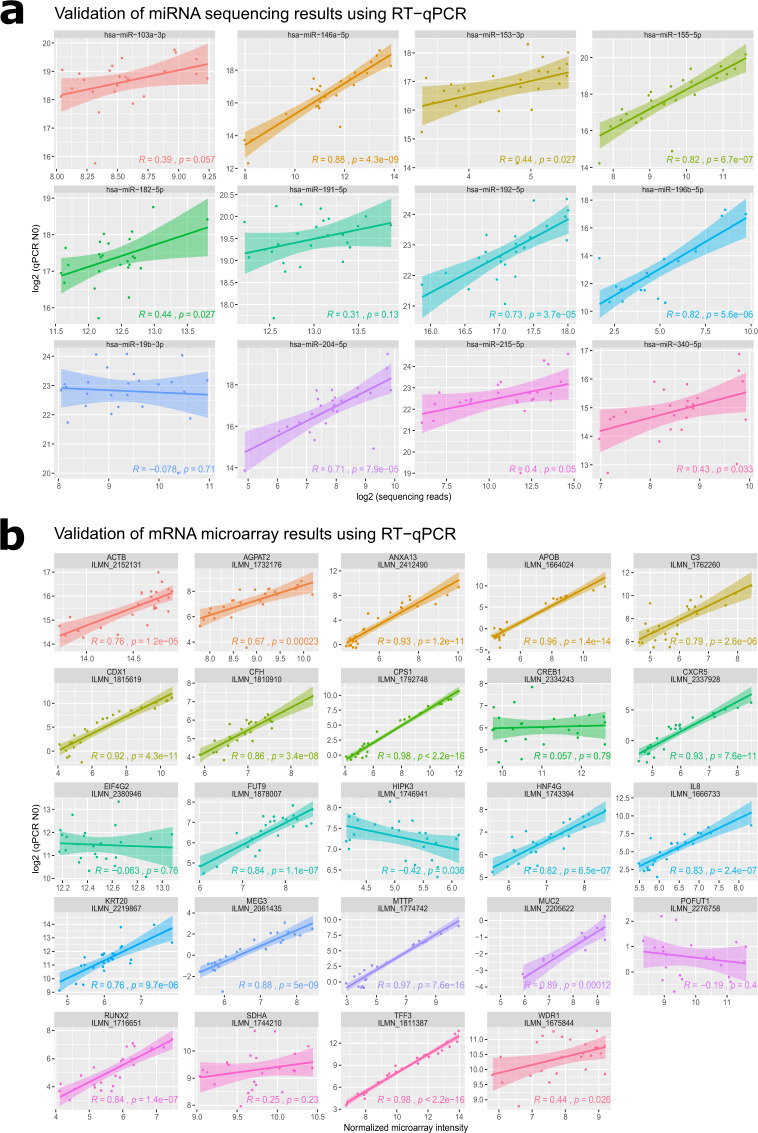
Validation of miRNAs (**a**) and messenger RNAs (**b**) by RT-qPCR. A panel 12 of miRNAs and 24 mRNAs were selected for validation of 25 RNA samples. Aliquots of the same RNA samples were used for sequencing, microarray and qPCR measurements. Raw qPCR data was exported to LinRegPCR software. N_0_ (an estimate of the target starting concentration per reaction) was calculated using the formula N_0_ = N_q_/E^Cq^ where E is the amplicon PCR efficiency and N_q_ is the fluorescence threshold set to determine C_q_. The Pearson correlation coefficient (*R*), the p-value and 95% confidence interval are indicated. Additional correlations to genes having multiple probes can be found in ref. ^29^.

To evaluate the concordance in gene expression between microarray or RNA-seq and qPCR, we calculated the correlation between normalized microarray/RNA-seq and qPCR log transformed N_0_ values (Fig. 4). Overall, high *R* and low p-values values (*R* > 0.8, p < 0.001) were observed between microarray and qPCR measurements. Some of them were probe dependent (i.e. C3 probe ILMN_1762260: *R* = 0.79, p < 0.001, but C3 ILMN_1662523 was not correlated). Five miRNA showed high correlation (*R* > 0.7, p < 0.001), 4 were poorly correlated (*R* ∼ 0.4, p < 0.05) and 3 were not correlated.

## Usage Notes

miRNA, small-RNA raw sequencing data (FASTQ) and normalized microarray data can be analysed by a variety of freely accessible packages and platforms, such as R/Bioconductor^27^. Some R/Bioconductor packages can be used without prior programming knowledge by using the Galaxy platform^28^.

The authors encourage proper citation of data sources for any work based on this dataset.

## Online-only Table

**Table-only Table 1:** Patient metadata for *H.pylori* infection.

Individual	Sample Name	Stomach location	Gender	Diagnosis	Age	*H. pylori* status	Intestinal metaplasia	Micro-array	Non-coding RNAseq	miRNA FASTQ file	SmallRNA FASTQ
patientB007	B007	antrum	F	AT	46	−	+	yes	yes	B007_miRNA_L007_R1.fastq.gz	B007_smallRNA_L007_R1.fastq.gz
patientB033	B033	antrum	F	IM	66	+	+	yes	yes	B033_miRNA_L001_R1.fastq.gz	B033_smallRNA_L001_R1.fastq.gz
patientB036	B036	antrum	M	IM	68	−	+	yes	yes	B036_miRNA_L002_R1.fastq.gz	B036_smallRNA_L002_R1.fastq.gz
patientB041	B041	antrum	F	IM	30	+	+	yes	yes	B041_miRNA_L003_R1.fastq.gz	B041_smallRNA_L003_R1.fastq.gz
patientB061	B061	antrum	F	AT	67	+	+	yes	yes	B061_miRNA_L004_R1.fastq.gz	B061_smallRNA_L004_R1.fastq.gz
patientB064	B064	antrum	F	IM	58	+	+	yes	yes	B064_miRNA_L005_R1.fastq.gz	B064_smallRNA_L005_R1.fastq.gz
patientB065	B065	antrum	F	IM	60	+	+	yes	yes	B065_miRNA_L006_R1.fastq.gz	B065_smallRNA_L006_R1.fastq.gz
patientB077	B077	antrum	F	IM	59	+	+	yes	yes	B077_miRNA_L007_R1.fastq.gz	B077_smallRNA_L007_R1.fastq.gz
patientB095	B095	antrum	F	AT	60	−	−	yes	yes	B095_miRNA_L003_R1.fastq.gz	B095_smallRNA_L003_R1.fastq.gz
patientB096	B096	antrum	M	AT	62	+	+	yes	yes	B096_miRNA_L008_R1.fastq.gz	B096_smallRNA_L008_R1.fastq.gz
patientB098	B098	antrum	F	IM	62	+	+	yes	yes	B098_miRNA_L008_R1.fastq.gz	B098_smallRNA_L008_R1.fastq.gz
patientB110	B110	antrum	M	AT	73	−	+	yes	yes	B110_miRNA_L006_R1.fastq.gz	B110_smallRNA_L006_R1.fastq.gz
patientB115	B115	antrum	M	IM	42	−	+	yes	yes	B115_miRNA_L001_R1.fastq.gz	B115_smallRNA_L001_R1.fastq.gz
patientB119	B119	antrum	F	IM	46	+	+	yes	yes	B119_miRNA_L001_R1.fastq.gz	B119_smallRNA_L001_R1.fastq.gz
patientB121	B121	antrum	M	AT	54	+	−	yes	yes	B121_miRNA_L001_R1.fastq.gz	B121_smallRNA_L001_R1.fastq.gz
patientB126	B126	antrum	M	IM	43	+	+	yes	yes	B126_miRNA_L002_R1.fastq.gz	B126_smallRNA_L002_R1.fastq.gz
patientB145	B145	antrum	F	IM	63	+	+	yes	yes	B145_miRNA_L003_R1.fastq.gz	B145_smallRNA_L003_R1.fastq.gz
patientB160	B160	antrum	F	IM	67	+	+	yes	yes	B160_miRNA_L002_R1.fastq.gz	B160_smallRNA_L002_R1.fastq.gz
patientB171	B171	antrum	M	IM	71	+	+	yes	yes	B171_miRNA_L004_R1.fastq.gz	B171_smallRNA_L004_R1.fastq.gz
patientB174	B174	antrum	F	IM	60	+	+	yes	yes	B174_miRNA_L003_R1.fastq.gz	B174_smallRNA_L003_R1.fastq.gz
patientB191	B191	antrum	M	IM	68	+	+	yes	yes	B191_miRNA_L004_R1.fastq.gz	B191_smallRNA_L004_R1.fastq.gz
patientB204	B204	antrum	M	IM	58	+	+	yes	yes	B204_miRNA_L005_R1.fastq.gz	B204_smallRNA_L005_R1.fastq.gz
patientB209	B209	antrum	F	AT	44	+	+	yes	yes	B209_miRNA_L006_R1.fastq.gz	B209_smallRNA_L006_R1.fastq.gz
patientB253	B253	antrum	M	IM	51	+	+	yes	yes	B253_miRNA_L007_R1.fastq.gz	B253_smallRNA_L007_R1.fastq.gz
patientB280	B280	antrum	F	IM	63	+	+	yes	yes	B280_miRNA_L008_R1.fastq.gz	B280_smallRNA_L008_R1.fastq.gz
patientB294	B294	antrum	M	IM	32	+	+	Not done	yes	B294_miRNA_L001_R1.fastq.gz	B294_smallRNA_L001_R1.fastq.gz
patientB308	B308	antrum	F	AT	49	+	−	yes	yes	B308_miRNA_L008_R1.fastq.gz	B308_smallRNA_L008_R1.fastq.gz
patientB311	B311	antrum	M	IM	28	+	+	Not done	yes	B311_miRNA_L005_R1.fastq.gz	B311_smallRNA_L005_R1.fastq.gz
patientB314	B314	antrum	M	IM	53	+	+	yes	yes	B314_miRNA_L005_R1.fastq.gz	B314_smallRNA_L005_R1.fastq.gz
patientB316	B316	antrum	F	IM	49	−	+	yes	yes	B316_miRNA_L006_R1.fastq.gz	B316_smallRNA_L006_R1.fastq.gz
patientB328	B328	antrum	M	IM	53	+	+	yes	yes	B328_miRNA_L003_R1.fastq.gz	B328_smallRNA_L003_R1.fastq.gz
patientB337	B337	antrum	M	IM	62	+	+	yes	yes	B337_miRNA_L004_R1.fastq.gz	B337_smallRNA_L004_R1.fastq.gz
patientB373	B373	antrum	M	IM	56	+	+	yes	yes	B373_miRNA_L002_R1.fastq.gz	B373_smallRNA_L002_R1.fastq.gz
patientB389	B389	antrum	F	IM	42	+	+	yes	yes	B389_miRNA_L006_R1.fastq.gz	B389_smallRNA_L006_R1.fastq.gz
patientB444	B444	antrum	M	IM	35	+	+	yes	yes	B444_miRNA_L007_R1.fastq.gz	B444_smallRNA_L007_R1.fastq.gz
patientB459	B459	antrum	M	IM	65	+	+	yes	yes	B459_miRNA_L007_R1.fastq.gz	B459_smallRNA_L007_R1.fastq.gz
patientB464	B464	antrum	F	IM	42	+	+	yes	yes	B464_miRNA_L008_R1.fastq.gz	B464_smallRNA_L008_R1.fastq.gz
patientB472	B472	antrum	F	IM	76	+	+	yes	yes	B472_miRNA_L008_R1.fastq.gz	B472_smallRNA_L008_R1.fastq.gz
patientB479	B479C	corpus	M	NAG	44	−	−	yes	yes	B479C_miRNA_L001_R1.fastq.gz	B479C_smallRNA_L001_R1.fastq.gz
patientB481	B481A	antrum	F	CAG	45	+	−	yes	yes	B481A_miRNA_L001_R1.fastq.gz	B481A_smallRNA_L001_R1.fastq.gz
patientB487	B487C	corpus	F	CAG	52	+	−	yes	yes	B487C_miRNA_L002_R1.fastq.gz	B487C_smallRNA_L002_R1.fastq.gz
patientB488	B488A	antrum	M	CAG	50	+	−	yes	yes	B488A_miRNA_L003_R1.fastq.gz	B488A_smallRNA_L003_R1.fastq.gz
patientB488	B488C	corpus	M	CAG	50	+	−	yes	yes	B488C_miRNA_L004_R1.fastq.gz	B488C_smallRNA_L004_R1.fastq.gz
patientB489	B489A	antrum	F	CAG	46	+	−	yes	yes	B489A_miRNA_L005_R1.fastq.gz	B489A_smallRNA_L005_R1.fastq.gz
patientB496	B496A	antrum	F	CAG	37	+	−	yes	yes	B496A_miRNA_L006_R1.fastq.gz	B496A_smallRNA_L006_R1.fastq.gz
patientB502	B502A	antrum	F	IM	62	+	+	yes	yes	B502A_miRNA_L001_R1.fastq.gz	B502A_smallRNA_L001_R1.fastq.gz
patientB502	B502C	corpus	F	CAG	62	+	−	yes	yes	B502C_miRNA_L002_R1.fastq.gz	B502C_smallRNA_L002_R1.fastq.gz
patientB504	B504A	antrum	F	CAG	55	+	−	yes	yes	B504A_miRNA_L007_R1.fastq.gz	B504A_smallRNA_L007_R1.fastq.gz
patientB504	B504C	corpus	F	CAG	55	+	−	yes	yes	B504C_miRNA_L008_R1.fastq.gz	B504C_smallRNA_L008_R1.fastq.gz
patientB505	B505A	antrum	F	IM	64	+	+	yes	yes	B505A_miRNA_L002_R1.fastq.gz	B505A_smallRNA_L002_R1.fastq.gz
patientB521	B521A	antrum	M	CAG	66	+	−	yes	yes	B521A_miRNA_L001_R1.fastq.gz	B521A_smallRNA_L001_R1.fastq.gz
patientB528	B528C	corpus	M	CAG	51	+	−	yes	yes	B528C_miRNA_L003_R1.fastq.gz	B528C_smallRNA_L003_R1.fastq.gz
patientB535	B535A	antrum	M	CAG	49	+	−	yes	yes	B535A_miRNA_L006_R1.fastq.gz	B535A_smallRNA_L006_R1.fastq.gz
patientB535	B535C	corpus	M	AT	49	+	−	yes	yes	B535C_miRNA_L002_R1.fastq.gz	B535C_smallRNA_L002_R1.fastq.gz
patientB551	B551A	antrum	F	IM	67	−	+	yes	yes	B551A_miRNA_L003_R1.fastq.gz	B551A_smallRNA_L003_R1.fastq.gz
patientB587	B587A	antrum	F	CAG	36	+	−	yes	yes	B587A_miRNA_L002_R1.fastq.gz	B587A_smallRNA_L002_R1.fastq.gz
patientB590	B590C	corpus	F	NAG	41	−	−	yes	yes	B590C_miRNA_L002_R1.fastq.gz	B590C_smallRNA_L002_R1.fastq.gz
patientB591	B591A	antrum	F	IM	48	+	+	yes	yes	B591A_miRNA_L004_R1.fastq.gz	B591A_smallRNA_L004_R1.fastq.gz
patientB591	B591C	corpus	F	IM	48	+	+	yes	yes	B591C_miRNA_L004_R1.fastq.gz	B591C_smallRNA_L004_R1.fastq.gz
patientB592	B592A	antrum	F	IM	69	+	+	yes	yes	B592A_miRNA_L005_R1.fastq.gz	B592A_smallRNA_L005_R1.fastq.gz
patientB592	B592C	corpus	F	CAG	69	+	−	yes	yes	B592C_miRNA_L005_R1.fastq.gz	B592C_smallRNA_L005_R1.fastq.gz
patientB593	B593A	antrum	F	CAG	63	+	−	yes	yes	B593A_miRNA_L003_R1.fastq.gz	B593A_smallRNA_L003_R1.fastq.gz
patientB598	B598A	antrum	M	NAG	46	−	−	yes	yes	B598A_miRNA_L003_R1.fastq.gz	B598A_smallRNA_L003_R1.fastq.gz
patientB598	B598C	corpus	M	NAG	46	−	−	yes	yes	B598C_miRNA_L004_R1.fastq.gz	B598C_smallRNA_L004_R1.fastq.gz
patientB599	B599A	antrum	M	NAG	68	−	−	yes	yes	B599A_miRNA_L005_R1.fastq.gz	B599A_smallRNA_L005_R1.fastq.gz
patientB599	B599C	corpus	M	NAG	68	−	−	yes	yes	B599C_miRNA_L006_R1.fastq.gz	B599C_smallRNA_L006_R1.fastq.gz
patientB601	B601A	antrum	F	AT	43	+	−	yes	yes	B601A_miRNA_L007_R1.fastq.gz	B601A_smallRNA_L007_R1.fastq.gz
patientB601	B601C	corpus	F	AT	43	+	−	yes	yes	B601C_miRNA_L008_R1.fastq.gz	B601C_smallRNA_L008_R1.fastq.gz
patientB605	B605A	antrum	F	AT	40	−	−	yes	yes	B605A_miRNA_L005_R1.fastq.gz	B605A_smallRNA_L005_R1.fastq.gz
patientB605	B605C	corpus	F	NAG	40	−	−	yes	yes	B605C_miRNA_L004_R1.fastq.gz	B605C_smallRNA_L004_R1.fastq.gz
patientB608	B608A	antrum	F	NAG	47	−	−	yes	yes	B608A_miRNA_L007_R1.fastq.gz	B608A_smallRNA_L007_R1.fastq.gz
patientB608	B608C	corpus	F	NAG	47	−	−	yes	yes	B608C_miRNA_L008_R1.fastq.gz	B608C_smallRNA_L008_R1.fastq.gz
patientB612	B612A	antrum	M	NAG	50	−	−	yes	yes	B612A_miRNA_L001_R1.fastq.gz	B612A_smallRNA_L001_R1.fastq.gz
patientB612	B612C	corpus	M	NAG	50	−	−	yes	yes	B612C_miRNA_L002_R1.fastq.gz	B612C_smallRNA_L002_R1.fastq.gz
patientB613	B613A	antrum	F	CAG	50	+	−	yes	yes	B613A_miRNA_L004_R1.fastq.gz	B613A_smallRNA_L004_R1.fastq.gz
patientB617	B617A	antrum	F	CAG	56	+	−	yes	yes	B617A_miRNA_L005_R1.fastq.gz	B617A_smallRNA_L005_R1.fastq.gz
patientB618	B618A	antrum	M	CAG	53	+	−	yes	yes	B618A_miRNA_L006_R1.fastq.gz	B618A_smallRNA_L006_R1.fastq.gz
patientB618	B618C	corpus	M	CAG	53	+	−	yes	yes	B618C_miRNA_L007_R1.fastq.gz	B618C_smallRNA_L007_R1.fastq.gz
patientB619	B619A	antrum	M	CAG	35	+	−	yes	yes	B619A_miRNA_L008_R1.fastq.gz	B619A_smallRNA_L008_R1.fastq.gz
patientB621	B621A	antrum	F	NAG	49	−	−	yes	yes	B621A_miRNA_L003_R1.fastq.gz	B621A_smallRNA_L003_R1.fastq.gz
patientB622	B622A	antrum	F	IM	66	+	+	yes	yes	B622A_miRNA_L006_R1.fastq.gz	B622A_smallRNA_L006_R1.fastq.gz
patientB624	B624C	corpus	F	CAG	61	+	−	yes	yes	B624C_miRNA_L001_R1.fastq.gz	B624C_smallRNA_L001_R1.fastq.gz
patientB625	B625A	antrum	F	NAG	47	−	−	yes	yes	B625A_miRNA_L004_R1.fastq.gz	B625A_smallRNA_L004_R1.fastq.gz
patientB629	B629A	antrum	F	AT	54	−	+	yes	yes	B629A_miRNA_L007_R1.fastq.gz	B629A_smallRNA_L007_R1.fastq.gz
patientB629	B629C	corpus	F	AT	54	−	+	yes	yes	B629C_miRNA_L008_R1.fastq.gz	B629C_smallRNA_L008_R1.fastq.gz
patientB634	B634C	corpus	M	AT	51	+	+	yes	yes	B634C_miRNA_L001_R1.fastq.gz	B634C_smallRNA_L001_R1.fastq.gz
patientB636	B636C	corpus	F	NAG	63	−	−	yes	yes	B636C_miRNA_L007_R1.fastq.gz	B636C_smallRNA_L007_R1.fastq.gz
patientB642	B642C	corpus	M	CAG	43	+	−	yes	yes	B642C_miRNA_L002_R1.fastq.gz	B642C_smallRNA_L002_R1.fastq.gz
patientB646	B646A	antrum	M	CAG	30	+	−	yes	yes	B646A_miRNA_L003_R1.fastq.gz	B646A_smallRNA_L003_R1.fastq.gz
patientB646	B646C	corpus	M	CAG	30	+	−	yes	yes	B646C_miRNA_L004_R1.fastq.gz	B646C_smallRNA_L003_R1.fastq.gz
patientB647	B647A	antrum	M	CAG	38	+	−	yes	yes	B647A_miRNA_L005_R1.fastq.gz	B647A_smallRNA_L003_R1.fastq.gz
patientB647	B647C	corpus	M	CAG	38	+	−	yes	yes	B647C_miRNA_L006_R1.fastq.gz	B647C_smallRNA_L003_R1.fastq.gz
patientB648	B648A	antrum	M	CAG	45	+	−	yes	yes	B648A_miRNA_L007_R1.fastq.gz	B648A_smallRNA_L003_R1.fastq.gz
patientB648	B648C	corpus	M	CAG	45	+	−	yes	yes	B648C_miRNA_L008_R1.fastq.gz	B648C_smallRNA_L003_R1.fastq.gz
patientB652	B652A	antrum	M	NAG	66	−	−	yes	yes	B652A_miRNA_L005_R1.fastq.gz	B652A_smallRNA_L003_R1.fastq.gz
patientB652	B652C	corpus	M	NAG	66	−	−	yes	yes	B652C_miRNA_L006_R1.fastq.gz	B652C_smallRNA_L006_R1.fastq.gz

F: female; M: male; NAG: non−active gastritis; CAG: chronic−active gastritis; AT: atrophic gastritis; IM: intestinal metaplasia; N/A: not available.
